# Efficacy of heel lifts versus calf muscle eccentric exercise for mid-portion Achilles tendinopathy (the HEALTHY trial): study protocol for a randomised trial

**DOI:** 10.1186/s13047-019-0325-2

**Published:** 2019-03-21

**Authors:** Chantel L. Rabusin, Hylton B. Menz, Jodie A. McClelland, Angela M. Evans, Karl B. Landorf, Peter Malliaras, Sean I. Docking, Shannon E. Munteanu

**Affiliations:** 1 Discipline of Podiatry, School of Allied Health, Human Services and Sport, La Trobe University, Melbourne, Victoria 3086 Australia; 2 Discipline of Physiotherapy, School of Allied Health, Human Services and Sport, La Trobe University, Melbourne, Victoria 3086 Australia; 3 La Trobe Sport and Exercise Medicine Research Centre, School of Allied Health, Human Services and Sport, La Trobe University, Melbourne, Victoria 3086 Australia; 4 Department of Physiotherapy, School of Primary and Allied Health Care, Faculty of Medicine, Nursing and Health Sciences, Monash University, Frankston, Victoria 3199 Australia

**Keywords:** Tendinopathy, Achilles tendon, Heel lift, Orthotic devices, Exercise therapy, Rehabilitation

## Abstract

**Background:**

Mid-portion Achilles tendinopathy is a common musculoskeletal condition characterised by degeneration of the Achilles tendon, which causes pain and disability. Multiple non-surgical treatments have been advocated for this condition including calf muscle eccentric exercise and in-shoe heel lifts. Although adherence is challenging, there is evidence to suggest that calf muscle eccentric exercise is effective in decreasing pain and improving function in people with Achilles tendinopathy. Heel lifts reduce ankle joint dorsiflexion and Achilles tendon strain, however their efficacy in the management of Achilles tendinopathy is unclear. This article describes the design of a parallel-group randomised trial comparing the efficacy of heel lifts to calf muscle eccentric exercise for Achilles tendinopathy.

**Methods:**

Ninety-two participants with Achilles tendinopathy will be randomised to one of two groups: (i) a heel lift group that will receive pre-fabricated 12 mm in-shoe heel lifts (Clearly Adjustable®), or (ii) an exercise group that will be advised to carry out a calf muscle eccentric exercise program (twice a day, 7 days a week, for 12 weeks). Outcome measures will be obtained at baseline, 2, 6 and 12 weeks; the primary endpoint for assessing efficacy being 12 weeks. The primary outcome measure will be the total score of the Victorian Institute of Sport Assessment – Achilles (VISA–A) questionnaire. Secondary outcome measures will include thickness and integrity of the Achilles tendon (using ultrasound tissue characterisation [UTC]), participant perception of treatment effect on pain and function (using the 7-point Patient Global Impression of Change scale), severity of pain at the Achilles tendon (using a 100 mm visual analogue scale) in the previous week, health status (using the EuroQol-5D-5L™ questionnaire), physical activity levels (using the 7-day Recall Physical Activity Questionnaire) and calf muscle function (using the standing heel rise test). Data will be analysed using the intention to treat principle.

**Discussion:**

The HEALTHY trial (Heel lifts versus calf muscle eccentric Exercise for AchiLles TendinopatHY) is the first randomised trial to compare the efficacy of heel lifts to calf muscle eccentric exercise in reducing pain and improving function in people with Achilles tendinopathy. A pragmatically designed trial was developed to ensure that if the interventions are found to be effective, the findings can be readily implemented in clinical practice.

**Trial registration:**

Australian New Zealand Clinical Trials Registry: ACTRN12617001225303. Registered on August 22nd, 2017.

**Electronic supplementary material:**

The online version of this article (10.1186/s13047-019-0325-2) contains supplementary material, which is available to authorized users.

## Background

Mid-portion Achilles tendinopathy is a common overuse soft tissue injury causing pain and stiffness with incidence rates reported to be 1.85 per 1000 people in the general population [1]. The majority of people with Achilles tendinopathy are active and involved in recreational or competitive sport [2]. Achilles tendinopathy is common in running populations with prevalence rates up to 9.5% [3] and a lifetime risk of 52% for top level (elite) runners [2]. However, the condition also affects inactive populations with 1 in 3 people with the condition leading a sedentary lifestyle [1]. Increased age is a contributing factor for the condition as it is reported most frequently by people aged between 40 and 59 years [4]. Obesity has also been associated with the condition [1]. People with Achilles tendinopathy typically report symptoms of pain and stiffness upon weight bearing after prolonged rest [5] and at the start of physical activity, which reduces as the activity continues [5]. These symptoms lead to impaired performance [6]. In more severe cases, pain and disability can be persistent with functional activities such as walking [7].

Numerous interventions have been advocated for the initial management of Achilles tendinopathy, including: exercise [8, 9], stretching [10], neuromuscular re-education [11], activity modification [12], patient counselling [12], in-shoe heel lifts [13], night splints [14], taping [15], low-level laser therapy [16], iontophoresis [17], dry needling [18], corticosteroids [19], and extracorporeal shockwave therapy [20]. However, the evidence supporting these interventions for the management of Achilles tendinopathy is equivocal [21]. When non-surgical interventions are ineffective, surgical management may be required, which is carried out in approximately 30% of people with the condition [6].

Exercise programs remain the most widely researched intervention for the management of Achilles tendinopathy [21, 22]. Multiple exercise programs have been described that include eccentric, concentric, isometric, and/or isokinetic contractions. Further, the proposed frequency, load, and speed of exercise programs have varied, and optimum exercise protocols are yet to be established [23]. For more than two decades, the eccentric calf muscle exercise protocol proposed by Alfredson [24] has been most frequently reported in literature and widely used by practitioners. The program involves three sets of 15 repetitions performed twice daily for 12 weeks with external resistance (as a percentage of body weight) added in the latter stages of the program with the aim to improve the tendon’s ability to withstand load. Although the program is inexpensive and evidence from systematic reviews and randomised trials have shown the eccentric exercise program decreases pain and improves function in people with Achilles tendinopathy [8, 25], the program is not without its limitations. The mechanism for its efficacy remains unclear and the program is time consuming. This may explain the variability in adherence [25–27], with the intervention reported to be unsuccessful in up to 44% of people with Achilles tendinopathy [20, 28]. A longer duration of symptoms, greater severity of the condition [28], and inactivity [28] have all been found to reduce the effectiveness of this intervention.

In-shoe heel lifts are another intervention advocated for the management of Achilles tendinopathy [29]. Heel lifts are inexpensive shoe inserts designed to place the ankle into a more plantarflexed position [30] and reduce Achilles tendon strain. Experimental studies have supported the use of heel lifts, showing reductions in ankle joint dorsiflexion during running [31], as well as reductions in Achilles tendon strain [32, 33], gastrocnemius activity [34], and peak force of posterior leg muscles [35] during walking. Despite this, there are only two low quality studies that have evaluated the efficacy of heel lifts for the management of Achilles tendinopathy. In a randomised trial, Lowdon and colleagues [36] evaluated the efficacy of rubber 15 mm heel lifts (Sorbothane® heel pads [*n* = 11], Molefoam heel pads [*n* = 10], and no heel pads [*n* = 12]) for “sports-induced Achilles tendinitis” in 33 participants aged 11 to 51 years. Participants who were randomised to receive heel lifts reported improvements in pain, swelling and tenderness associated with Achilles tendinopathy at two months, however there were no between-group comparisons. In addition, a case series [29] evaluated the benefits of heel lifts for “Achilles tendonitis” in 14 participants. At three months, the majority (11/14) of participants reported returning to activity pain free. Although this was a favourable outcome, this study had a small sample size, no control group, and the heel lift height was unspecified. Clearly, there is insufficient evidence to demonstrate the effectiveness of heel lifts in the management of Achilles tendinopathy.

Given the limitations of the available evidence, the aim of this randomised trial is to evaluate the efficacy of heel lifts versus calf muscle eccentric exercise for Achilles tendinopathy.

## Methods

This trial protocol has been reported in accordance with the Standard Protocol Items: Recommendations for Interventional Trials (SPIRIT) guidelines [37]. The SPIRIT checklist is included in an additional file [see Additional file 1].

### Design

**Fig. 1:**
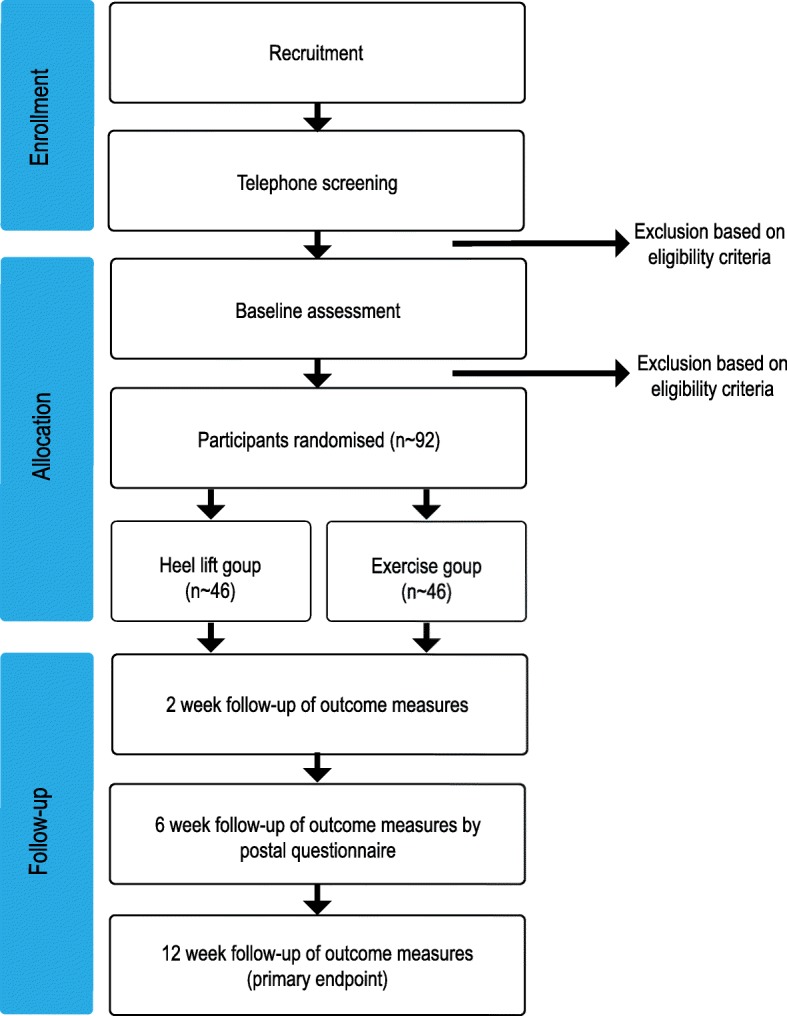
Study protocol

The HEALTHY study (Heel lifts versus calf muscle eccentric Exercise for AchiLles TendinopatHY) is a parallel-group randomised superiority trial (Fig. 1) with a 12-week follow up. Participants will be randomised to receive one of two interventions: (i) Clearly Adjustable® pre-fabricated in-shoe heel lifts, or (ii) a calf muscle eccentric exercise program. All participants will receive an intervention, therefore there are no ethical concerns of not treating participants experiencing pain.

Due to the nature of the intervention, research staff administering the interventions (i.e. the therapists) and assessing outcomes (i.e. the assessors) will not be blinded to group allocation. Similarly, it is not possible to blind the participants. However, research staff entering and analysing data will be blinded.

Assessments will occur at baseline (initial), 2, 6 and 12 weeks. Assessments will be performed at the La Trobe University Health Sciences Clinic, Melbourne (Victoria, Australia) with the exception of the 6 week assessment (postal questionnaire).

### Ethics approval

Ethical approval has been obtained from the La Trobe University Human Ethics Committee (Application No HEC17–064). Publications associated with the trial will be reported according to the Consolidated Standards of Reporting Trials (CONSORT) 2010 Statement [38, 39]. The trial has been registered with the Australian New Zealand Clinical Trials Registry (ACTRN 12617001225303). Prior to involvement in this trial, all participants will review the Participant Information Statement form and provide written/electronic informed consent. Ethical standards will adhere to the National Health and Medical Research Council (NHMRC) National Statement [40].

### Participant recruitment

Participants will be recruited by advertisements placed in relevant local newspapers and magazines (Melbourne, Australia), by posters placed in local community sporting clubs and universities (Melbourne, Australia), mail-out advertisements to healthcare practitioners in Melbourne, and by a mail-out to people currently accessing podiatry services at the La Trobe University Health Sciences Clinic. In addition, we will advertise this trial using social media (such as Twitter, Facebook and Instagram).

### Eligibility criteria

Telephone screening by one of the authors (CLR) will assess the suitability of participants for this trial. Suitable participants will then attend an initial assessment for further eligibility screening.

#### Inclusion criteria

To be included in this trial, participants must satisfy the below criteria:

(i) aged 18 years or greater;
(ii) Achilles tendon symptoms (pain) present in one or both lower limb(s) for a minimum of two months;
(iii) report having pain rated at least 3 out of 10 on a numerical rating scale (NRS-11);
(iv) be literate in English and able to complete the questionnaires used in this trial (such as the primary outcome measure, the Victorian Institute of Sport Assessment – Achilles [VISA–A] questionnaire);
(v) regularly use footwear that can accommodate a heel lift of up to 12 mm height;
(vi) be able to walk household distances (more than 50 m) without the aid of a walker, crutches or cane;
(vii) be willing to attempt to not use additional treatments (such as shoe modifications, physiotherapy, foot orthoses/bracing, injections, or surgery) for the Achilles pain during the trial (12 weeks);
(viii) be willing to attempt to discontinue taking all pain-relieving medications to relieve pain for the Achilles tendon(s) (except paracetamol) for at least 14 days prior to the initial assessment and during the trial (12 weeks);
(ix) be willing to attend the Health Sciences Clinic at La Trobe University (Melbourne, Victoria) for an assessment on three occasions.

Achilles tendinopathy will be diagnosed based on a clinical assessment and musculoskeletal ultrasound [41–43] using the following criteria:

(i) insidious onset of pain in the region of the Achilles tendon, aggravated by weight bearing activity;
(ii) pain located 2 to 6 cm proximal to the Achilles tendon insertion upon palpation by the investigator;
(iii) gray-scale musculoskeletal ultrasound of the Achilles tendon showing diffuse or local thickening with or without irregular fibre orientation and hypoechoic areas within the mid-portion of the Achilles tendon [44].

#### Exclusion criteria

Participants will be excluded from this trial if they have any of the criteria listed below [20, 25, 28]:

(i) currently pregnant;
(ii) previous Achilles tendon surgery in the symptomatic lower limb(s);
(iii) previous Achilles tendon rupture in the symptomatic lower limb(s);
(iv) chronic ankle instability (at least one significant ankle sprain, a history of an injured ankle that “gave way”, recurrent ankle sprains or ankle instability) [45];
(v) conditions of the Achilles tendon/ankle region that are not Achilles tendinopathy such as ankle osteoarthritis, impingement syndrome, insertional Achilles tendinopathy, Achilles paratenonitis;
(vi) inflammatory arthritis (e.g. ankylosing spondylitis);
(vii) metabolic or endocrine disorders (e.g. type I or II diabetes);
(viii) neurological disorders (e.g. Charcot-Marie-Tooth disease);
(ix) previous breast cancer and/or use of oestrogen inhibitors;
(x) treatment with heel lifts or calf muscle eccentric exercise within the previous three months;
(xi) use of fluoroquinolone antibiotics within the previous two years;
(xii) injection of local anaesthetic, corticosteroid or other pharmaceutical agent into the Achilles tendon or surrounding area within the previous three months;
(xiii) any medical condition that, in the opinion of the investigators, makes the participant unsuitable for inclusion (e.g. clinically important pain in the musculoskeletal system other than the Achilles tendon);
(xiv) cognitive impairment (defined as a score of < 7 on the Short Portable Mental Status Questionnaire) [46].

### Baseline assessment

#### Participant characteristics and anthropometrics

Structured questionnaires will be used to obtain data regarding the presentation of symptoms (lower limb[s] affected, location, characteristics, duration of symptoms, current treatment), levels of physical activity (type, frequency, duration), medical conditions (medical history, surgical history, current medications), use of mobility aids/orthoses and current footwear (style, age and frequency of use). Demographic and anthropometric data will be collected (such as age, sex, weight, height, hip and waist circumference). Height and weight will be measured using a stadiometer and digital scales, and body mass index will be calculated as weight (kg)/height (m^2^) and waist to hip ratio will be calculated as waist circumference (cm)/hip circumference (cm). Static foot posture will be assessed using the Foot Posture Index [47]. Participants’ shoe size will also be documented, and their footwear will be assessed using selected items from the Footwear Assessment Tool [48]. Ankle joint dorsiflexion range of motion will be measured using a reliable weightbearing lunge technique [49, 50]. Participants will be informed that they have been randomly allocated to receive either heel lifts or an exercise program. Prior to randomisation, and to determine participant preference for the interventions, participants will be asked ‘if you had a choice, which group would you prefer to be allocated to and why?’ [51].

#### Musculoskeletal ultrasound assessment

**Fig. 2:**
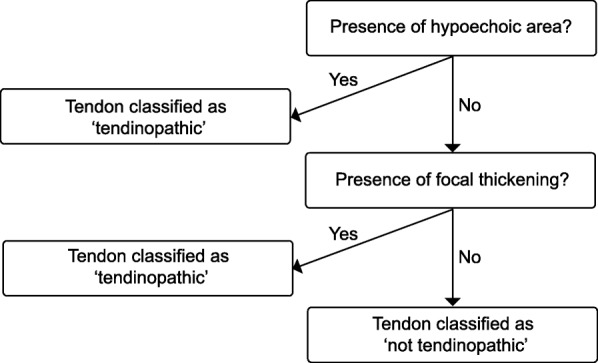
Algorithm for classification of Achilles tendinopathy using UTC

Participants will undergo musculoskeletal ultrasound using ultrasound tissue characterisation (UTC) imaging of the Achilles tendon by a registered podiatrist (CLR) at baseline (initial) and 12 weeks. An algorithm will be used to classify the Achilles tendon as normal or pathological (Fig. 2). Participants who display either local thickening [42], irregular fibre orientation, or irregular tendon structure with hypoechoic areas within the mid-portion of the Achilles tendon [52] will be diagnosed with Achilles tendinopathy [20]. Certain features have been shown to concomitantly exist in those with Achilles tendinopathy, and may also exist in asymptomatic individuals [43]. Therefore, participants will not be excluded if they have aforementioned sonographic features accompanied by fluid in the retrocalcaneal bursae (up to 4.0 mm), focal calcifications, paratenon thickening (considered to be present if the paratenon measures more than 2.0 mm in thickness) [43] or calcaneal cortical anomalies (such as spurring).

### Interventions

#### Random allocation and concealment

Participants will be randomised to one of two groups: (i) a heel lift group (pre-fabricated heel lifts [Clearly Adjustable®]), or (ii) an exercise group (calf muscle eccentric exercise program). To ensure allocation concealment, a cloud-based randomisation service provided by Griffith University, Queensland, Australia, will be used. The random allocation sequence will be generated with a 1:1 allocation ratio using permuted blocks of random sizes.

Interventions will be administered by CLR, a registered podiatrist with five years clinical experience. Prior to the trial, CLR will be mentored and undergo training sessions by the senior members of the research team who have extensive expertise in the assessment and management of musculoskeletal disorders including Achilles tendinopathy.

#### In-shoe heel lifts (heel lift group)

**Fig. 3:**
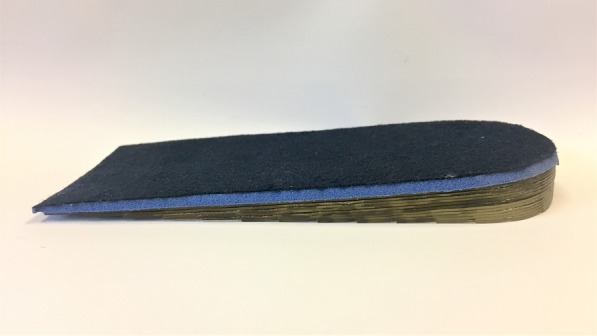
Clearly Adjustable® heel lifts

Participants randomised to the heel lift group will receive Clearly Adjustable® (Algeos Australia) 12 mm in-shoe heel lifts for both feet. The heel lifts are made of firm multi-layered clear vinyl. Three sizes will be available (small, medium and large) and will be issued according the participants’ shoe size. The Clearly Adjustable® in-shoe heel lifts will have a 3.2 mm PPT® Ultralux top cover applied (Fig. 3) to the upper surface to maximise comfort. Three sets of heel lifts will be dispensed to participants to be worn in up to three of their most frequently worn shoes. Participants will be advised to remove any existing ‘insoles’ from their shoes and to wear the heel lifts as much as possible whilst wearing shoes. The maximum heel lift height (12 mm) will be used, however the height of the heel lifts will be reduced if required (and documented) to fit in the participants’ shoes without causing heel slippage during gait. A handout providing instructions about using the heel lifts will be provided to participants.

#### Calf muscle eccentric exercise (exercise group)

**Fig. 4:**
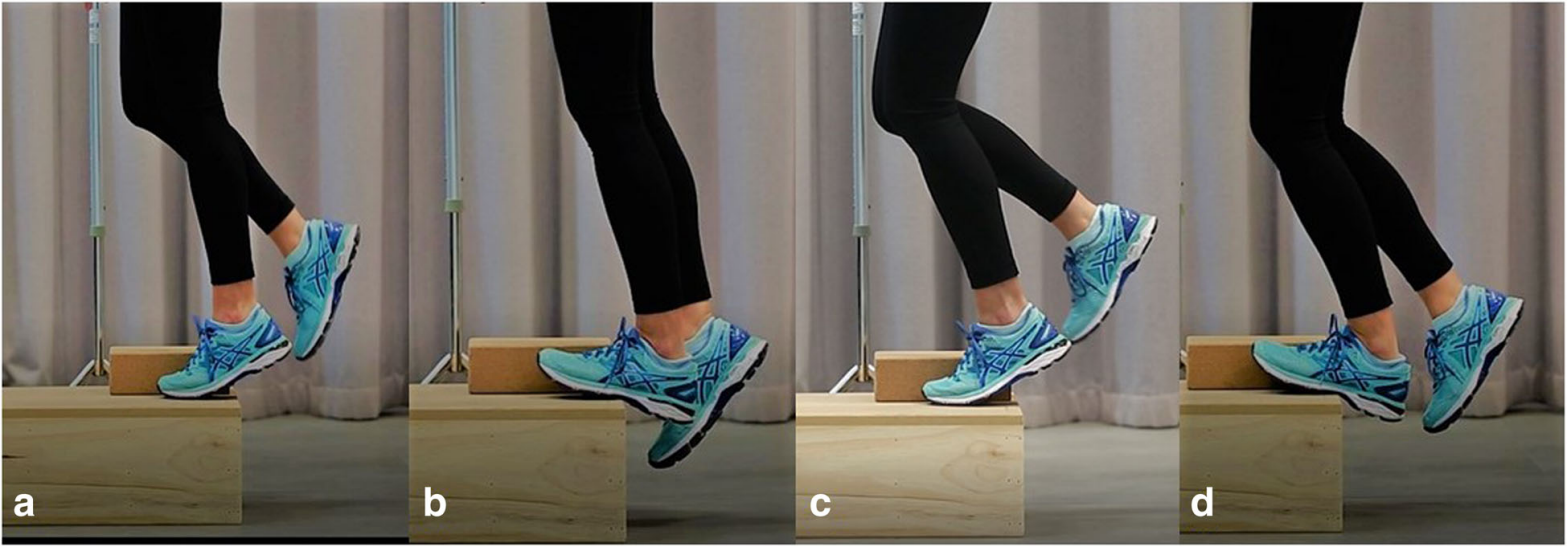
Calf muscle eccentric exercise program. Figures **a** and **b** show (**a**) the start position and (**b**) end position of the straight-knee exercise. Figures **c** and **d** show (**c**) the start position and (**d**) end position of the bent-knee exercise. Note the use of a block to aid the contralateral leg in lifting the patient

Participants randomised to the exercise group will receive a calf muscle eccentric exercise program based on the Alfredson [24] method. Participants will be provided with a handout and video explaining and demonstrating how to perform the exercise program. Each participant will require a small step (at least 20 cm from the ground) to perform this program. Participants will be instructed to perform eccentric calf muscle exercises to below plantigrade (toes on a step) twice daily, 7 days/week for 12 weeks. In participants with bilateral symptoms, the exercise program will be performed on both sides. From an upright body position and standing with all bodyweight on the forefoot and the ankle joint in plantarflexion, the calf muscle will be loaded eccentrically by having the participant lower the heel beneath the forefoot. Participants will avoid concentric loading of the Achilles tendon of the ipsilateral lower limb by placing a 10 cm high block under the foot of the contralateral lower limb to allow them to lift themselves back to the start position and minimise any concentric contraction of the calf muscle of the contralateral limb – this is important for participants with bilateral Achilles tendinopathy. This exercise will be performed in two positions: (i) with the knee extended, and (ii) with the knee flexed to maximise the activation of the soleus muscle. Both types of calf muscle eccentric loading exercises will involve three sets of 15 repetitions. Participants with bilateral Achilles tendinopathy will be instructed to perform the eccentric exercises on both lower limbs (one at a time) (Fig. 4).

Initially, resistance will consist of bodyweight, and the participants will stand with all their bodyweight on the injured leg. Once participants can perform both exercises with no pain or discomfort on the injured side, they will increase the resistance component of the program. Participants will be asked to use a weighted back-pack to increase resistance. Participants will begin by adding 5 kg of mass (e.g. bags of sand, books) to the backpack and then perform both exercises (3 sets of 15 repetitions) twice daily. Participants will be advised to increase the load by 5 kg increments.

All participants will be advised that muscle soreness within the first two weeks is to be expected. Participants will be advised to continue the exercise program even if pain presents, however if at any point the pain becomes disabling during the exercise program participants will be advised to stop. Participants will also be advised to apply ice over and around the Achilles tendon for 15 min following completion of each exercise session.

### Modification of physical activity

Both groups will also receive a modified physical activity program based on the pain-monitoring model [53]. This approach has been shown to be safe and produces comparable outcomes to programs that involve complete rest in individuals with Achilles tendinopathy [53]. Our research team has also used this modified program effectively in a previous clinical trial [25]. Participants will be allowed to partake in their usual activities (rather than complete rest) after receiving the intervention, provided the amount of pain they experience in the Achilles tendon pain does not exceed level 5 on a 0–10 pain scale, where 0 is no pain and 10 is worst pain imaginable during exercise/activity. The pain after usual physical activities can reach a 5 on the pain scale, but should subside the following morning. During activity, if the pain in the Achilles tendon exceeds 5 on the pain scale participants will need to reduce their activity/exercise (if possible).

### Treatment credibility/expectation

Outcomes of interventions can be affected by participants expectations (how much participants expect they will benefit from the treatment) and credibility (participants’ beliefs about the logic of an intervention) [54]. Given that participants will not be blinded to their allocated intervention, determining their beliefs about treatment is important. With this in mind, participants will complete the Credibility/Expectancy Questionnaire (CEQ) [55] after being allocated to an intervention group. The CEQ contains 6 items that ask the participants to rate the credibility of the intervention and their expectations on a 9-point Likert scale. Higher scores on the scale suggest the participant considers the intervention to be logical and convincing (credible) and improvements will occur with the intervention (expectancy). The CEQ has been shown to be reliable (good internal consistency and test-retest reliability) [55].

### Outcome measures

The primary outcome will be measured at baseline (prior to randomisation) and at 12 weeks (the primary end-point). All secondary outcomes unless stated otherwise will be measured at baseline (prior to randomisation) and at 2, 6 and 12 weeks (primary end-point). Participants will be encouraged to contact the researchers at any time during the trial.

#### Primary outcome measure

The primary outcome measure for this trial will be the total score of the Victorian Institute of Sport Assessment – Achilles (VISA–A) questionnaire, developed to primarily assess the clinical severity of Achilles tendinopathy [56]. The VISA–A questionnaire, is a quick, self-administered questionnaire that has been validated and shown to have good test-retest reliability in this population [56]. The VISA–A contains 8 questions that evaluate pain, function and activity in patients with Achilles tendinopathy. Seven questions are scored out of 10 (questions 1 to 7) and one question is scored out of 30 (question 8), with a maximum calculated score of 100 being obtainable. The greater the VISA–A score the less severe the Achilles tendinopathy. A limitation of the VISA–A [56] is that it is primarily designed for sporting populations, yet Achilles tendinopathy also occurs in non-sporting populations. Specifically, question 8 of the VISA–A uses the word ‘sports’, thus making the question irrelevant to non-sporting populations. For this trial, we have replaced the word ‘sport’ with ‘physical activity’ to ensure question 8 is relevant to both sporting and non-sporting populations. This amendment has also been used previously by our research team in a clinical trial [25]. Participants with bilateral symptoms will be asked to describe symptoms based on the most painful lower limb (or the right lower limb if they cannot define the most painful lower limb).

#### Secondary outcome measures

Secondary outcome measures include: (i) thickness and integrity of the Achilles tendon, (ii) participant perception of treatment effect, (iii) severity of pain, (iv) health status, (v) level of physical activity in the previous week and (vi) calf muscle function. Secondary outcome measures are described in more detail below.

(i) Thickness and integrity of the Achilles tendon: ultrasound tissue characterisation (UTC) will be used to visualise the Achilles tendon structure (including thickness) and assess tendon integrity. Participants will be positioned prone, with feet overhanging the examination table and in a neutral position to examine the tendon and paratendinous structures in the transverse and longitudinal planes [43]. A high resolution probe positioned in the UTC tracking device will move automatically along the long axis of the Achilles tendon. The UTC device will be positioned on the posterior surface of the Achilles tendon parallel to the tendon’s long axis, at the Achilles insertion (proximal aspect of the calcaneus). Scanning will be conducted proximal to distal. The maximum anterior-posterior tendon thickness will be measured using electronic callipers [43, 53].

The tendon structure of each participant will be quantified by percentage of the four echo-types as described by Van Shie et al. [57]:

- Echo-type I: intact, continuous and aligned fibres and fasciuli;
- Echo-type II: less continuous and/or more wavy fibres and fasciuli;
- Echo-type III: mainly fibrillary matrix;
- Echo-type IV: complete disintegration, with tendon tissue replacement by an amorphous matrix and fluid.

To quantify tendon structure, contours will be manually placed around the Achilles tendon in the transverse view at regular intervals along the length of the Achilles tendon (disappearance of the calcaneus to the musculotendinous junction) [44]. The UTC software (UTC2010, UTC Imaging) automatically interpolates between the contours to create a 3-D volume where proportions of each echo type are calculated. The percentage of echo type I + II (structure-related) and echo type III and IV (non-structure related) will be then be calculated. The percentage of normal tendon (i.e. echo types I and II) will then be determined from this data [57].

(ii) Participant perception of the treatment effect: the participant’s perception of treatment effect will be measured separately for pain and function using the Patient Global Impression of Change (PGIC) questionnaire [58]. The scale includes the following questions for participants: ‘Compared to how you were before starting this trial, how is your pain in your Achilles tendon?’, and ‘Compared to how you were before starting this trial, how would you rate your ability to perform physical activities (such as walking, dancing, running, gardening, housework)?’. Each question will have the following responses ‘very much improved’, ‘much improved’, ‘minimally improved’, ‘no change’, ‘minimally worse’, ‘much worse’, or ‘very much worse’. The responses will then be dichotomised according to ‘treatment effectiveness’ where ‘treatment effectiveness’ is defined as ‘much improved’ or ‘very much improved’ [59].

(iii) The severity of pain: the severity (intensity) of pain will be measured using a 100 mm VAS, where participants will be asked to, ‘Mark on the line the severity of pain you have experienced in your Achilles tendon when it has been at its worst, over the past week’. Zero (0) implies no pain and one hundred (100) implies the worst pain imaginable [58].

(iv) Health status: EuroQol (EuroQol 5D-5 L®), a standardised questionnaire for describing health-related quality of life, will be used to measure health status [60]. This measurement tool consists of five questions addressing mobility, self-care, usual activities, pain/discomfort, and anxiety/depression. The participant will also rate their overall health state from 0 (worst health state imaginable) to 100 (best imaginable health state) using a VAS.

(v) Level of physical activity in the previous week: the level of physical activity in the previous week will be evaluated using the 7-day Recall Physical Activity Questionnaire, a valid and reliable tool [61]. This questionnaire has been used previously as an outcome measure in clinical trials of interventions for lower limb musculoskeletal pathologies including Achilles tendinopathy [25, 59].

(vi) Calf muscle function: calf muscle function will be measured at baseline and at 12 weeks using the standing heel rise test [62]. This test involves participants using their fingertips lightly placed on a wall for balance, whilst minimising postural sway, standing on their affected side and then repetitively raising onto their toes (moving the ankle into plantarflexion) until fatigue. This test will be performed to the sound of a metronome (one heel rise every 2 s with a metronome set at 60 beats per minute). The maximum number is then recorded. This test has been previously used and shown to have adequate reliability (ICC = 0.61 to 0.98) in both healthy participants [63] and participants with Achilles tendinopathy [64, 65]

#### Use of co-interventions to relieve pain at the Achilles tendon

The use of paracetamol rescue medication and co-interventions to relieve pain at the Achilles tendon will be measured at 2, 6 and 12 weeks via questionnaires. Participants will be asked to document any new treatment that they have used to relieve pain in the Achilles tendon. This includes modifications to footwear usually worn, foot orthotics/modifications, use of taping/bracing, visits to general practitioners, medical specialists, other healthcare practitioners such as physiotherapists or podiatrists, use of any creams/rubs or taken any pain relieving medication such as paracetamol or anti-inflammatory drugs. Participants will be asked to document the type of treatment, the date they received the treatment and, if relevant, dosage.

### Evaluation of adherence

Adherence will be measured at 2, 6 and 12 weeks via questionnaires. For the heel lift group, participants will provide information regarding the average number of hours per day and number of days they have worn the heel lifts in the last 2, 4 and 6 weeks respectively. For the exercise group, participants will provide information regarding the average number of days per week they have performed their exercises in the last 2, 4 and 6 weeks respectively. A recall of the previous weeks rather than daily dairy entries will minimise participant burden [66].

### Adverse events

Adverse events will be assessed at 2, 6 and 12 weeks via a questionnaire. Participants will be asked if they have experienced any new pain or injuries (ache, discomfort or stiffness) in the past 2, 4 or 6 weeks that has lasted for one day or longer. Participants will be asked to describe the location (region and side), severity (mild, moderate or severe) and duration (days, weeks) of symptoms [66]. If participants experience severe symptoms they will be asked to contact one of the investigators. All adverse events will be included in the final manuscript.

**Fig. 5:**
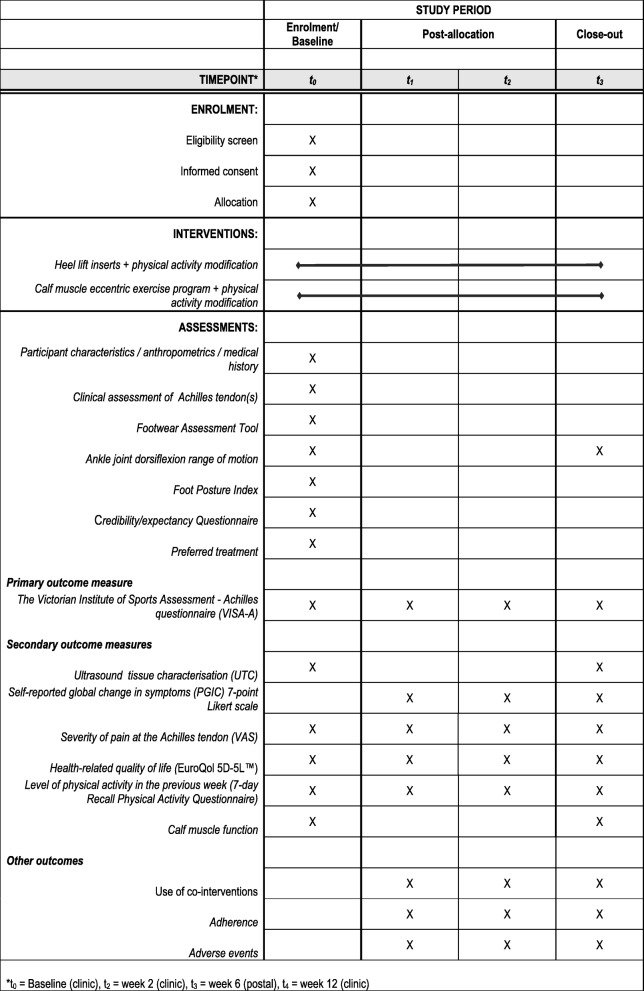
Data collection time points

The summary of the data collection time points for each outcome measure is shown in Fig. 5.

### Sample size

The sample size has been determined a priori using the t-test for two independent groups with common variance function in SPSS Sample Power 3.0 (IBM Corporation, USA) based on the VISA–A questionnaire as the primary outcome. Using an allocation ratio of 1:1, a power of 80%, minimal important difference (MID) of 10 points [28], standard deviation of 16.9 [25] (standardised effect size = 0.59) and alpha set at 0.05, we estimate that a minimum of 92 participants (i.e. approximately 46 per group) will be required. We did not allow for participant loss to follow-up in our calculation as missing data will be imputed [67]. Further, we have conservatively ignored the extra precision provided by covariate analysis when estimating the sample size.

### Data monitoring

A Data Monitoring and Ethics Committee (DMEC) will not be required for this study. This study is relatively short and has included two safe and commonly used interventions for participants who are not considered to be vulnerable [68, 69]. This study will have a Trial Management Committee that will comprise of senior study investigators (SEM, HBM, KBL, AME, JAM and PM). The Committee will meet every two weeks to review safety reports, data quality, protocol adherence and retention rates.

### Statistical analysis

The most recent version of SPSS (IBM Corp., Armonk, NY, USA) will be used to perform statistical analysis. The intention-to-treat principle will be used for all randomised participants. Where participants have bilateral symptoms, the more painful side will be analysed (or the right side if they cannot define the more painful side) to maintain independence of data. Multiple imputation will be used to replace any missing data using five iterations, with sex, age, baseline scores, and group allocation as predictors [67]. However, data substitution will not be applied for the following variables; self-reported magnitude of symptom change, use of co-interventions, adherence and adverse events. Standard tests to assess continuous data for normal distribution (such as assessing skewness and kurtosis) will be used and transformation carried out if required.

The primary outcome measure will be the VISA–A questionnaire measured at 12 weeks. To avoid over-testing and to minimise the risk of type I error associated with serial measurements, statistical analysis of the efficacy of the interventions will specifically focus on the change in primary outcome measures between baseline and 12 weeks [70, 71], and differences in the primary and secondary outcome measures between the two groups will be compared at 12 weeks. To minimise regression to the mean, continuously-scored outcome measures will be analysed using analysis of covariance (ANCOVA) with baseline scores, and intervention group entered as independent variables [72]. Relative risk, risk difference, and number needed to treat (NNT) will be used to compare dichotomous-scaled outcome measures. To complement point estimates, 95% confidence intervals and *p*-values will be calculated where appropriate.

## Discussion

Achilles tendinopathy is a common musculoskeletal condition causing pain and disability in both sporting and non-active populations. With the increasing number of older and overweight people in the population, the burden of the condition is likely to grow. Eccentric calf muscle loading programs have been the most widely researched and recommended intervention for the management of Achilles tendinopathy [24]. However, this intervention is not without its limitations, the program is demanding and adherence can be challenging [25, 28]. The program has also been found to be less effective in non-active populations, and where the condition is chronic and severe [20, 28]. Therefore, there is a need to evaluate other potentially effective interventions. One such intervention that has been advocated for the management of Achilles tendinopathy is heel lifts. Although evidence exists that shows heel lifts reduce Achilles tendon strain [32] and reduce ankle joint dorsiflexion [31], which suggests that this intervention may be therapeutically beneficial for Achilles tendinopathy, only two low quality studies [29, 36] have evaluated their efficacy for this condition.

Our parallel-group superiority trial with two active treatment groups will be the first randomised trial to compare the efficacy of heel lifts to a calf muscle eccentric exercise program in the management of Achilles tendinopathy. Although we considered including a non-active intervention arm (such as a wait-and-see group) in our protocol, we decided against this as it would not be ethical to withhold treatment as there is already a known effective treatment in the form of a calf muscle eccentric exercise program [73]. We have included a widely used outcome measure as the primary outcome (VISA–A) to allow us to compare the results across other studies that have investigated interventions for Achilles tendinopathy. We have also included a number of secondary outcomes to allow us to capture the effects of interventions on a wide variety of clinically relevant measures. This includes structural integrity of the Achilles tendon, the global rating of change in pain and function, physical activity levels and calf muscle function.

Recruitment commenced in August 2017, and we expect that the results will be finalised by September 2019. Results from this trial will provide clinically relevant information that will assist clinicians in the management of mid-portion Achilles tendinopathy.

## Additional file

Additional file 1: The SPIRIT checklist for the HEALTHY trial. (DOCX 60 kb)

## Abbreviations

BMI: Body mass index
CONSORT: Consolidated Standards of Reporting Trials
FPI: Foot Posture Index
MSKUS: Musculoskeletal ultrasound
NHMRC: National Health and Medical Research Council
PGIC: Patient Global Impression of Change
UTC: Ultrasound tissue characterisation
VAS: Visual Analogue Scale
VISA–A: Victorian Institute of Sport Assessment-Achilles
